# Interaction Between Glaucoma and Central Retinal Vein Occlusion in a Cohort Study

**DOI:** 10.3390/jcm14238472

**Published:** 2025-11-28

**Authors:** Abdullah Amini, Mette Bertelsen, Anne-Sofie Petri, Allan Linneberg, Henrik Vorum, Michael Larsen

**Affiliations:** 1 Department of Ophthalmology, Rigshospitalet, Copenhagen University Hospital, 2600 Glostrup, Denmarklars.michael.larsen@regionh.dk (M.L.); 2 Faculty of Health and Medical Sciences, University of Copenhagen, 2200 Copenhagen, Denmark; 3 Department of Medical Genetics, Rigshospitalet, 2100 Copenhagen, Denmark; 4 Center for Clinical Research and Prevention, Copenhagen University Hospital, Bispebjerg and Frederiksberg, 2000 Frederiksberg, Denmark; 5 Department of Ophthalmology, Aalborg University Hospital, 9000 Aalborg, Denmark

**Keywords:** central retinal vein occlusion, glaucoma, cataract, venous congestion, capillary leakage, venous pressure, retinal perfusion

## Abstract

**Objectives**: To study the associations of central retinal vein occlusion (CRVO) with glaucoma and cataract before and after the onset of CRVO. **Methods**: This study included 439 fundus photographically verified CRVO cases and a 5:1 set of 2195 registry-based age- and sex-matched control subjects without a record of CRVO. The study assessed rates of cataract and glaucoma before and after CRVO based on diagnoses, procedures, and prescriptions and analyzed their association with CRVO. Odds ratio (OR) and incidence rate ratio (IRR) estimates for 10 years prior to a subject’s first CRVO and incident comorbidity after CRVO were compared. **Results**: The median age at the time of presentation of 439 eligible patients with CRVO was 71 years (interquartile range 11 years). In the 10 years leading up to the incidence of CRVO, the ORs for glaucoma and cataract were 6.01 (95% confidence interval (CI95) 4.05 to 8.94) and 2.13 (CI95 1.45 to 3.12), respectively. During a mean follow-up of 5.7 years after CRVO, the incidence rate ratios for glaucoma and cataract were 16.7 (CI95 9.32–30.1) and 1.99 (CI95 1.39–2.84), respectively. **Conclusions**: Glaucoma and cataract occurred at elevated rates compared with the background population, both before and after the clinical presentation of CRVO. The results fit a disease model where retinal perfusion is compromised by chronic venous congestion, leading to glaucomatous retinal degeneration. Chronic venous congestion may subsequently convert to clinically manifest CRVO when retinal capillaries have been sufficiently weakened to produce hemorrhage, edema and vision loss.

## 1. Introduction

Central retinal vein occlusion (CRVO) can present with variable degrees of venous congestion, retinal and optic disc hemorrhage, retinal ischemia, and macular edema [1,2]. The congestion is objectively detectable in the retina, but no clinically available method can determine the location of the occlusion, the degree to which the occlusion is caused by compression or thrombosis, the venous pressure or its development over time. Central retinal vein occlusion can have an insidious onset, over months or years [3], and the clinical manifestations are dominated by a mixture of retinal hemorrhage, edema, and ischemia, which are products of leakage as much as of venous congestion [1,4]. This means that retinal perfusion pressure may have been subnormal for some time before the appearance of CRVO. Central retinal vein occlusion is epidemiologically intertwined with glaucoma, the presence of one being associated with a high risk of having or developing the other. Glaucoma can be preceded by stromal hemorrhage on and around the optic disc [5,6,7,8]. The study hypothesis was that an element of venous congestion may be involved in some types of glaucoma or conditions that are classified as glaucoma. In this fundus photography and health registry study, we examined rates of glaucoma and cataract before and after CRVO and compared rates of glaucoma and cataract in CRVO patients with those in the background population.

## 2. Materials and Methods

This was a registry-based matched case–control and cohort study including 439 verified CRVO cases assessing ocular risk factors for CRVO with follow-up data from three secondary referral centres, the Rigshospitalet and the Aalborg and Odense hospitals in Denmark, between 1976 and 2010. Reference data were obtained from a national database and comprised 5 optimally matching individuals without a diagnosis of CRVO for every case of CRVO [9]. The study was approved by the Committee on Health Research Ethics of the Capital Region of Denmark (jr.no. F-24045982) and conducted in accordance with the Declaration of Helsinki II.

Fundus images, fluorescein angiograms and medical records of patients diagnosed with CRVO (code H.348 of the International Classification of Disease 10 and codes 37703, 37708, and 37709 of the International Classification of Disease 8) were reviewed to confirm the diagnosis, which required the presence of disc swelling, macular edoema, dilated retinal veins, intraretinal hemorrhage in all four fundus quadrants, and cotton wool spots. In chronic cases, it was accepted that congestion, edema, hemorrhage, and cotton-wool spots were absent, provided chronic changes were present, such as collaterals around the optic disc. Also accepted were concurrent diabetic or diabetic retinopathy-like microvascular abnormalities, if they were present in both eyes [10]. Patients with multiple CRVO events were included based on their initial CRVO event. The patients were aged 40 years or older.

Control subjects were selected from the general population in the Danish Civil Registration System (DCRS), which has maintained essential information on all citizens in Denmark since 1968 using unique personal identity numbers [11]. Five unduplicated same-sex control subjects per case (2195 in total) were automatically selected at random among subjects who were eligible based on their age being within ±2 years of the case, being of the same sex, not having a diagnosis of CRVO, and being alive on the day CRVO was diagnosed in the case.

Comorbidity data were evaluated using information from the Danish National Patient Registry (DNPR) and the Registry of Medicinal Product Statistics (RMPS). The DNPR, established in 1977, encompasses public and private hospital contacts and associated diagnoses in Denmark, using ICD-8 until 1995 and ICD-10 from 1995 [12]. Since 1995, the RMPS has documented all prescription drug dispensations at pharmacies in Denmark by civil registration number, date, and type of drug (Anatomical Therapeutic Chemical code) [13].

Chronic conditions were classified using hospital discharge diagnoses and drug prescriptions. The presence of glaucoma was inferred if antiglaucoma eye drop medication had been dispensed, and so on. Isolated events were based on hospital discharge diagnoses. Cataract was assigned if cataract surgery had been registered. Glaucoma and cataract were the only ocular associations that were deemed to be of sufficient prevalence and registry-based diagnostic reliability to be included in the analysis. Registration was per patient, not per eye. The data were not of sufficient granularity to reliably define the sidedness of conditions, to differentiate between ocular hypertension and glaucoma, or to differentiate between glaucoma subtypes.

Survival data and migration status up to 31 December 2010 were obtained from the DCRS. Comorbidity data, collected until 31 December 2010, were accessible from 1968 (DCRS), 1977 (DNPR), and 1994 (RMPS), respectively. The study period was divided into the decade preceding the diagnosis of CRVO and the interval between the CRVO diagnosis and the date of censorship. Patients were censored upon the initial occurrence of an incident within a specified category, at the time of their death, or at the end of follow-up on 31 December 2010, whichever occurred first. Distinct analyses were conducted for each of the two periods. Only patients with a comprehensive set of registry data for a specified period were incorporated into the study of that period.

### Statistics

Data on diagnosis, gender, date of birth, index date (date of diagnosis, in patients), age at index date, observation duration, and outcome events were recorded for each patient and control subject. To identify missing data and extreme values, the data set was systematically tested. No extreme values were detected. Minor data deficiencies were identified among the socioeconomic factors, occurring in equal proportions in both cases and controls. Deletion of missing data or categorization as unknown did not change the estimates. Hence, no changes were made to the data set in terms of deletion or addition.

The study population was categorized into five age groups at the index date: younger than 50 years, 50–59 years, 60–69 years, 70–79 years, and older than 80 years. Conditional logistic regression was used to evaluate socioeconomic characteristics (income, education, and employment) as potential confounders.

Conditional regression analysis was used to obtain odds ratios (ORs) and 95% confidence intervals (95% CI) for each potential risk factor during the 10-year period preceding the CRVO. The ORs were adjusted for age at the index date (the matched variable), gender, index date, and socioeconomic characteristics (income, education, and employment) as categorical variables. Systematic testing did not identify any interactions.

Cox’s proportional hazard regression was used to estimate hazard ratios (HRs) and 95% confidence intervals (CI95), which were interpreted as a measure of incidence rate ratios (IRRs) and used to compare event rates between groups. Initially, the model was constructed using each covariate considered to be potentially influential. Time was used as the underlying scale in the final model, and IRRs were adjusted based on the index date by gender, age, and socioeconomic characteristics. Systematic testing did not identify any interactions.

Data were linked using population-wide unique personal identification numbers and anonymized before being made available for analysis using SAS (version 9.2, SAS Institute, Inc., Cary, NC, USA) on terminals linked to Statistics Denmark.

A prior report about the cohort showed that CRVO was associated with higher mortality than in the background population, and extraocular risk factors and events associated with CRVO [9]. The current report was made using shared data, with ocular morbidity constituting the principal subject of the study.

## 3. Results

The 439 CRVO cases (52% males, 48% females), most of whom were in the age range 60–79 years when diagnosed with CRVO, had socioeconomic characteristics comparable to those of the control group. The mean follow-up was 5.1 years after CRVO and 5.7 years for control subjects (Table 1).

Risk factors for CRVO recorded during the decade leading up to the CRVO at *p* < 0.05 in a multifactorial analysis were, in order of decreasing relative impact, glaucoma, diabetes with end-organ damage, peripheral artery disease, lymphoma, peripheral venous disease, cataract, diabetes, arterial hypertension, cardiovascular disease, cerebrovascular disease, and ischemic heart disease (Table 2).

Excessive morbidity after the CRVO at *p* < 0.05 in a multifactorial analysis was, in order of decreasing relative impact, glaucoma, peripheral venous disease, congestive heart failure, cerebrovascular disease, myocardial infarction, ischemic heart disease, peripheral artery disease and cataract (Table 2).

## 4. Discussion

This study found that the risk of developing CRVO was increased in people with preexisting systemic conditions such as peripheral vascular disease, ischemic and congestive heart disease, cerebrovascular disease, and diabetes, and with preexisting ocular conditions. Glaucoma was associated with a 6-fold increase in the risk of CRVO and cataract, associated with more than 2-fold elevation of the risk of CRVO (Table 2).

Clinical methods of investigation do not enable direct analysis of the mechanisms whereby the systemic risk factors promote congestion and occlusion of the central retinal vein. Their general characteristics suggest that combinations of thrombophilia, hyperviscosity, systemic venous congestion, arteriosclerosis, and cardiac insufficiency can induce thrombus formation in the central retinal vein, within or behind the lamina cribrosa. The same systemic risk factors may promote glaucoma and cataract, but they could also be linked to CRVO by shared exposure to adverse intraocular conditions. Of note, most cases of CRVO show preserved perfusion of the central retinal vein at the lamina cribrosa, implying that no categorical shift from open to closed has taken place. Furthermore, insidious onset of CRVO is commonly seen in clinical practice, beginning with a few retinal hemorrhages, progressing slowly to overt CRVO with macular edema [3,14]. This may be interpreted as a process of gradual occlusion by thrombosis or compression, but it could also result from the gradual weakening of the retinal vessel walls in response to preexisting subclinical venous congestion. The existence of a self-reinforcing mechanism that can abruptly tip into a clinical crisis is supported by the impressive therapeutic effect of bolus administration of intravitreal VEGF inhibitors, whose intravitreal concentrations can persist for multiple half-lives beyond the duration of a therapeutically meaningful drug concentration [15,16]. An added effect of presymptomatic venous congestion will be that of reducing retinal perfusion pressure, potentially inducing retinal hypoxia and loss of substance in the inner and middle layers of the retina [5,6,7,8], similar to the manner in which venous congestion can lead to local aggravation of diabetic retinopathy [3,17]. The existence of such a silent risk factor for retinal vascular disease is also suggested by the 8% prevalence of retinal microaneurysms and hemorrhages in people without diabetes [10].

Examination of the spontaneous pulsation of the large retinal veins suggests that the venous pressure can vary from being near the intraocular pressure in eyes with spontaneous venous pulsation on the optic disc to near the diastolic arterial blood pressure in eyes with angiographic venous backflow during the cardiac diastole [18]. Thus, eyes with CRVO show no venous pulsation on the optic disc [19], whereas venous pulsation is seen in 75–98% of healthy subjects and in 51–64% of glaucoma patients [20,21,22]. Furthermore, ophthalmodynamometry has shown that CRVO are associated with a higher-than-normal retinal venous pressure [23,24].

Our results are in agreement with and add substantial numerical power to prior studies of the association between glaucoma and CRVO [25,26,27,28,29,30,31,32,33]. Of particular value is the demonstration of glaucoma preceding CRVO, which is more intriguing than glaucoma coming after CRVO, when ocular ischemia may induce neovascularization of the trabecular meshwork.

The rate of bilaterality of CRVO at presentation has been found to be 1.6% [34], and the conversion rate from unilateral CRVO to bilateral unspecified RVO is only 3.4–4.4% per year over 3 years [34,35]. The predominant unilaterality of CRVO suggests a strong role for random intraocular risk factors [4], a key component of which may be variation in vessel distribution on and around the optic disc.

The effect of intravitreal pharmaceutical inhibition of vascular endothelial growth factor (VEGF) on macular edema and retinal vascular leakage secondary to CRVO is profound, supporting that relapse of CRVO is driven by vessel wall frailty rather than recurrent thrombosis of the central retinal vein. Of note, the suppression of macular edema can last for many months [15], despite the intravitreal drug half-life being only 7 days [16]. This supports the idea that VEGF inhibition allows leaking retinal vessels to regain structural integrity and withstand high venous pressure, a healing response that outlasts the intraocular lifetime of the VEGF inhibitor.

The elevated risk of CRVO following cataract surgery may be mediated by inflammation induced by cataract surgery and the associated promotion of blood-retina barrier leakage [36,37]. The elevated risk of cataract after CRVO may hypothetically be related to the same hypoxia that provokes anterior segment neovascularization.

The study is limited by the range of potential risk factors that can be pulled from available data sources, many of which have been established for administrative purposes, by the diagnosis of glaucoma being based on prescription data for pressure-lowering eye drops, by lack of registration of glaucoma subtype, ocular hypertension, anterior chamber neovascularization [38], and the laterality of CRVO and glaucoma. The latter may have led to overestimation of the odds ratios for glaucoma and cataract [23,24]. The study is also limited by a lack of detailed information about the types of glaucoma that drive the association between glaucoma and subsequent CRVO.

The data are from before the introduction of intravitreal VEGF-inhibition therapy for CRVO and are therefore free of the confounding effects of such interventions. Studies from the current era may find a lower incidence of neovascular glaucoma after CRVO.

Our observations of a link between glaucoma and subsequent CRVO fit a model in which constitutively elevated retinal venous pressure confers an elevated risk of symptomatic retinal vascular leakage, retinal hemorrhage, and macular edema. Theoretically, such subclinical venous pressure elevation will reduce retinal perfusion pressure and may exhaust vasomotor mechanisms that help maintain balanced blood flow throughout the retina. Future studies should examine this hypothesis and assess whether unevenness of retinal perfusion may help explain why glaucoma is associated with an increased risk of CRVO.

## Figures and Tables

**Table 1:** Demographic data for central retinal vein occlusion cases and control subjects, matched by sex and age.

	CRVO Cases (%) (*n* = 439)	Controls (%) (*n* = 2195)
Age [y] *		
<50	55 (12.5)	273 (12.4)
50–59	60 (13.7)	304 (13.8)
60–69	111 (25.3)	554 (25.2)
70–79	139 (31.7)	679 (31.1)
≥80	74 (16.9)	385 (17.5)
Gender		
Male	230 (52.4)	1150 (52.4)
Female	209 (47.6)	1045 (47.6)

CRVO = Central retinal vein occlusion. * at the time of each CRVO index case diagnosis. Income, employment and education characteristics were comparable between cases and controls as previously reported [9].

**Table 2:** Risk factors for central retinal vein occlusion 10–0 years before occlusion and risk factors for incident comorbidity after occlusion.

	10-Year Period Before CRVO				After CRVO			
	Cases (%)	Controls (%)	Odds Ratio (95% CI) *	*p*-Value	Cases (%)	Controls (%)	IRR (95%CI) **	*p*-Value
Myocardial infarction	29 (6.74)	97 (4.51)	1.54 (0.97–2.43)	0.06	19 (4.99)	67 (3.41)	2.16 (1.27–3.67)	0.004
Congestive heart failure	21 (4.88)	76 (3.53)	1.39 (0.83–2.34)	0.21	36 (9.23)	106 (5.35)	2.64 (1.78–3.91)	<0.0001
Ischemic heart disease	60 (13.1)	210 (9.77)	1.50 (1.08–2.08)	0.01	38 (10.80)	146 (7.88)	2.08 (1.44–3.02)	0.0001
Peripheral artery disease	35 (7.64)	62 (2.88)	3.51 (2.23–5.53)	<0.0001	18 (4.81)	58 (2.90)	2.01 (1.14–3.52)	0.01
Peripheral venous disease	13 (3.02)	32 (1.49)	2.26 (1.15–4.43)	0.02	15 (3.78)	31 (1.53)	3.52 (1.84–6.73)	0.0001
Cerebrovascular disease	48 (11.16)	148 (6.88)	1.68 (1.16–2.44	0.002	50 (13.70)	158 (8.26)	2.26 (1.63–3.15)	<0.0001
Liver disease	7 (1.63)	17 (0.70)	2.28 (0.83–6.24)	0.09	-	-	-	-
Lymphoma	8 (1.86)	13 (0.60)	3.03 (1.14–8.03)	0.02	-	-	-	-
Renal disease	6 (1.40)	24 (1.12)	1.06 (0.39–2.92)	0.90	-	-	-	-
Diabetes with end-organ damage	26 (6.05)	1 (0.05)	3.83 (2.26–6.49)	<0.0001	-	-	-	-
Hypercoagulability	0 (0.00)	1 (0.05)	-	-	-	-	-	-
Diabetes ^†^	42 (16.2)	113 (8.73)	2.07 (1.39–3.08)	0.0003	14 (5.09)	46 (3.15)	1.83 (0.98–3.39)	0.07
Arterial hypertension ^†^	178 (68.7)	719 (55.5)	2.02 (1.46–2.78)	<0.0001	34 (28.6)	236 (27.3)	1.37 (0.94–2.00)	0.10
Cardiovascular disease ^†‡^	197 (76.1)	846 (65.3)	1.88 (1.35–2.64)	0.002	38 (33.9)	269 (33.3)	1.31 (0.92–1.89)	0.14
Glaucoma ^†^	62 (23.9)	72 (5.56)	6.01 (4.05–8.94)	<0.0001	34 (13.9)	21 (1.38)	16.7 (9.32–30.1)	<0.0001
Cataract	48 (11.16)	137 (6.37)	2.13 (1.45–3.12)	0.0001	42 (11.6)	150 (7.81)	1.99 (1.39–2.84)	0.0002

CRVO = Central retinal vein occlusion. CI = Confidence interval. * Logistic regression estimating odds ratio (OR) adjusted for age, gender, index date and socioeconomic characteristics to evaluate the risk factors for CRVO. ** Cox regression model estimating incidence rate ratio (IRR) with time as underlying timescale adjusted for sex, age, index date and socioeconomic characteristics as categorical variables. ^†^ Diagnoses based on both hospital discharge diagnoses and drug prescriptions (The remaining diagnoses are based on hospital discharge diagnoses only). ^‡^ Cardiovascular disease includes hypertension, cerebrovascular disease, ischemic heart disease, congestive heart failure, peripheral vascular disease and cardiovascular drugs.

## Data Availability

The source data are curated by Danmarks Statistik.

## Abbreviations

The following abbreviations are used in this manuscript:

CRVO	Central retinal vein occlusion
OR	Odds ratio
IRR	Incidence rate ratio
CI	Confidence interval
DCRS	Danish Civil Registration System
DNPR	Danish National Patient Registry
RMPS	Registry of Medicinal Product Statistics
ICD	International Classification of Diseases
HR	Hazard ratio
VEGF	Vascular endothelial growth factor
